# Sustained Release of Protein Therapeutics from Subcutaneous Thermosensitive Biocompatible and Biodegradable Pentablock Copolymers (PTS*gels*)

**DOI:** 10.1155/2016/2407459

**Published:** 2016-10-05

**Authors:** Elizabeth Schaefer, Santhi Abbaraju, Mary Walsh, Donna Newman, Jacklyn Salmon, Rasidul Amin, Sidney Weiss, Ulrich Grau, Poonam Velagaleti, Brian Gilger

**Affiliations:** ^1^North Carolina State University, Raleigh, NC, USA; ^2^Symmetry Biosciences, Research Triangle Park, NC, USA; ^3^i-novion, Inc., Randolph, NJ, USA

## Abstract

*Objective*. To evaluate thermosensitive, biodegradable pentablock copolymers (PTS*gel*) for sustained release and integrity of a therapeutic protein when injected subcutaneously.* Materials and Methods*. Five PTS*gels* with PEG-PCL-PLA-PCL-PEG block arrangements were synthesized.* In vitro* release of IgG from PTS*gels* and concentrations was evaluated at 37°C. Released IgG integrity was characterized by SDS-PAGE.* In vitro* disintegration for 10GH PTS*gel* in PBS was monitored at 37°C over 72 days using gravimetric loss and GPC analysis. Near-infrared IgG in PTS*gel* was injected subcutaneously and examined by* in vivo* imaging and histopathology for up to 42 days.* Results*. IgG release was modulated from approximately 7 days to more than 63 days in both* in vitro* and* in vivo* testing by varying polymer composition, concentration of PTS*gel* aqueous solution, and concentration of IgG. Released IgG* in vitro* maintained structural integrity by SDS-PAGE. Subcutaneous PTS*gels* were highly biocompatible and* in vitro* IgG release occurred in parallel with the disappearance of subcutaneous gel* in vivo*.* Conclusions*. Modulation of release of biologics to fit the therapeutic need can be achieved by varying the biocompatible and biodegradable PTS*gel* composition. Release of IgG parallels disappearance of the polymeric gel; hence, little or no PTS*gel* remains after drug release is complete.

## 1. Introduction

Increase in the use of biologics has occurred over the past decade for the treatment, prevention, or cure of a variety of diseases in humans. Biological products include blood-derived products, vaccines, immunoglobulins, cells or microorganisms, and other proteins [1–4]. These have led to major therapeutic advances in several prevalent diseases, including immune-mediated arthritis and cancer immunotherapy [5]. Many biologics are administered to the patient, usually by daily or weekly subcutaneous injection. A controlled, sustained release therapeutic would decrease the frequency of injections, leading to increased patient compliance and therapeutic efficacy.

Sustained release subcutaneous therapeutics have been available for several decades, but recent advances in polymer science have led to development of hydrogels that provide sustained drug release, have high tissue biocompatibility, and allow self-administration by the patient [6]. Hydrogels provide a deformable drug depot that slowly elutes a high concentration of drug to surrounding tissue for an extended period of time [6]. However, because most hydrogels only physically incorporate, instead of forming covalent bonds to the drugs, a rapid drug release occurs over a few hours to days, limiting their value for sustained drug delivery [6].

Triblock copolymers of poly(ethylene oxide)-poly(propylene oxide)-poly(ethylene oxide) (PEO-PPO-PEO, poloxamers/pluronics) are the most widely used reverse thermal gelation polymers [7]. Other types of multiblock amphiphiles (i.e., polymers with both hydrophilic and hydrophobic domains) have been synthesized using a wide range of polymers. Some of these hydrogels are sufficiently deformable to be injectable, but many are not, necessitating surgical implantation for drug delivery. In either case, a high initial burst and lack of sustained drug release limit the clinical utility of these hydrogels [6, 8].

Polylactic-co-glycolic acid (PLGA) based hydrogels exhibit better biodegradability, higher gelation temperatures (permitting easier handling before injection), and longer periods of sustained drug release compared to poloxamer systems [9]. However, degradation of PLGA and PLGA copolymers produces lactic acid and glycolic acid, which reduces local pH substantially and may degrade protein therapeutics [10]. Furthermore, local tissue reaction to the PLGA may reduce tolerability and biocompatibility [11]. Therefore, an injectable and biocompatible hydrogel that provides a sustained release of biologically active protein therapeutic remains to be developed.

Pentablock copolymers are thermosensitive gels (*PTSgel*) composed of biodegradable or biocompatible polymer blocks, such as polyethylene glycol (PEG), polycaprolactone (PCL), polyglycolide (PGA), and polylactic acid (PLA). The relative block arrangement and molecular weight of PTS*gel* polymers affect the solution-gelation (sol-gel) transition behavior, degradation, and* in vitro* release characteristics of the hydrogel [12]. PTS*gels* may act as a drug delivery vehicle by entrapping the drug in the core of a micelle of PTS*gel* [12]. PTS*gels* can be injected through a small-gauge needle to form a firm,* in situ*, hydrogel depot [12]. PTS*gels* have been demonstrated to be biocompatible* in vitro* and* in vivo* and provide sustained release of immunoglobulin G (IgG) [12, 13]. Furthermore, enhanced stability of biologic proteins (IgG and bevacizumab) delivered from PTS*gels in vitro* was recently shown [13]. The amounts of PLA used in the described polymers ranged from 28 to 37% of the total molar mass. Compared to PLGA, the lower molar mass of PLA or PGA blocks in the PTS*gel* produces much lower amounts of lactic acid or glycolic acid on degradation, thereby improving protein stability of the delivered biologic. Therefore, the potential advantages of PTS*gels* as carriers for subcutaneous sustained delivery of protein biologic therapeutics include biodegradation, their high biocompatibility, long-term release kinetics, ease of injectability, and stability of the protein therapeutic being delivered.

The objective of this work was to further evaluate the sustained release properties of promising thermosensitive PTS*gels* for the controlled release of a model full-length therapeutic protein (IgG; mw 150 kDal) for subcutaneous injection. This study investigated the* in vitro* modulated release of IgG, the structural integrity of released IgG, and the* in vivo* duration of IgG release from PTS*gel* after subcutaneous injection.* In vitro*/*in vivo* correlation has been established and presented for selected PTS*gel* polymers. The study also investigated* in vitro* disintegration of 10GH PTS*gel* in PBS (pH 7.4) at 37°C over a period of several weeks.

## 2. Materials and Methods

### 2.1. PTS*gel *Synthesis

Monomethoxy PEG (550), L-lactide, tin octoate, hexamethylenediisocynate (HMDI), ethylene glycol-bis(2-aminoethylether)-N,N,N′,N′-tetraacetic acid (EGTA), sodium sulfate, dichloromethane, heptanes, and endotoxin free water were purchased from Sigma-Aldrich (St. Louis, MO). *ε*-Caprolactone was purchased from Alfa Aesar (Ward Hill, MA). PTS*gels* with PEG-PCL-PLA-PCL-PEG block arrangements were synthesized as previously described [12, 13]. Briefly, the diblock copolymer was synthesized by ring-opening copolymerization of *ε*-caprolactone with monomethoxy PEG using tin octoate as a catalyst. The resulting diblock copolymer was similarly converted to triblock by adding L-lactide. The resulting triblock copolymer was coupled utilizing hexamethylenediisocynate (HMDI) as a linker to prepare PEG-PCL-PLA-PCL-PEG pentablock copolymers. The purified pentablock was stored at −20°C until being used.

### 2.2. PTS*gel *Characterization

#### 2.2.1. Fourier Transform Infrared Spectroscopy (FTIR) Analysis

Fourier transform infrared spectroscopy (FTIR) spectra were recorded with a Perkin Elmer Spectrum Version 10.03.09 infrared spectrophotometer. FTIR scan of neat polymer was carried out in a range of 4000–400 cm^−1^.

#### 2.2.2. ^1^H-NMR Analysis

Purity, molecular structure, and molecular weight (Mn) of the PTS*gel* were analyzed utilizing a Mercury 300 MHz NMR spectrometer. ^1^H-NMR spectrograms were recorded by dissolving the polymers in deuterated chloroform (CDCl_3_).

#### 2.2.3. Gel Permeation Chromatography (GPC) Analysis

Molecular weights (Mn and Mw) and polydispersity of polymers were examined by GPC analysis. Briefly, 20 mg of polymer was dissolved in 1 mL of tetrahydrofuran (THF). Polymer samples were separated on two OligoPore columns (Agilent, Santa Clara, CA) connected in series and maintained at 40°C. Solvent THF at the rate of 0.6 mL/min was utilized as eluting solvent. Samples were analyzed on Wyatt technologies MINI DAWN instrument (S. number 528-T) connected to OPTILAB DSP interferometric refractometer, using ASTRA 6 software.

#### 2.2.4. Determination of Tin, Organic Solvents, and Endotoxin Levels in the Polymers

Amount of stannous (Tin) present was determined on 5% aqueous polymer solution using ICP-OES method on a Thermo iCAP 6100 instrument. Residual dichloromethane and heptane in the PTS*gel* were determined by head space GC-MS using an Agilent 5890 GC and an Agilent 5972 mass spectrometer. The column employed was Restek Rtx-5MS, 30 m × 0.25 mm, 0.1 *μ*m film. The endotoxin levels were determined using Pierce Chromogenic Endotoxin Quantification Kit (Thermo Fisher Scientific, Waltham, MA).

### 2.3. Sol-Gel Transition Testing at 37°C

The sol (flow)-gel (no flow) transition of PTS*gel* was examined by following a previously published protocol [14]. Briefly, the polymers were dissolved in PBS buffer (pH 7.4) at 25 wt% concentration. 0.5 mL of aqueous polymeric solution was transferred into 2.5 mL glass vial and placed in water bath maintained at 37°C. Vials were kept for 5 min at 37°C. Gel formation was observed visually by inverting the tubes, immediately after pulling out of the water bath.

### 2.4. *In Vitro *PTS*gel *Disintegration (Incubation in PBS Buffer, pH 7.4 at 37°C)

#### 2.4.1. Gravimetric Measurement of the Residual Gelling Polymer

Three concentrations (12.5, 18.75, and 25%) of PTS*gel* 10GH were evaluated for disintegration/degradation* in vitro*. Each individual PTS*gel* (500 *μ*L of 10GH in triplicate) was pipetted into an 8 mL glass vial, weighed, and placed into a 37°C water bath for gelling for 30 minutes. 4 mL of PBS (37°C) with 0.02% sodium azide as preservative was placed over each PTS*gel*. The vials were placed into a 37°C shaker water bath, maintained at 60 rpm. Every 5 days, vials were centrifuged (2000 rpm for 5 minutes at 37°C), the PBS buffer was removed and replaced with fresh PBS, and the vials returned to the 37°C shaker water bath. Samples of gel were withdrawn after 0, 5, 15, 30, and 45 days and every 15 days thereafter until polymer completely disintegrated. At each time point, vials with residual gel were stored at −80°C until being lyophilized. Vials containing lyophilized gels were weighed and the dry gel weight was determined by subtracting the empty vial weight from the final dry weight.

#### 2.4.2. GPC Analysis of the Residual Gelling Polymer and the Supernatant

The residual gelling polymer (PTS*gel* 10GH) and the supernatant collected during the gravimetric studies were analyzed using GPC. The gels were lyophilized using a Labconco Model 5 lyophilizer (Kansas City, MO) and 10–50 mL of the buffer samples were dried using a Genevac EZ-2 evaporator overnight. The lyophilized and dried samples were dissolved in 1 mL of tetrahydrofuran and further dried using 0.3–0.5 g of sodium sulfate. Samples were then vortexed and filtered using 0.2 *μ*m PTFE ISO disc filters. Selected samples were analyzed by GPC as described in Section 2.2.3.

### 2.5. *In Vitro *Release of IgG from PTS*gel*

*In vitro* drug release experiments were conducted by adding 500 *μ*L of 22.5% aqueous gelling polymeric solution containing 10 mg of IgG (Human IgG, Lee Biosolutions, Maryland Heights, MO) into an 8 mL silanized glass vial (Thermo Scientific, Waltham, MA) in triplicate. Vials were incubated in a 37°C water bath for ~5 minutes until polymer gelled. Release buffer, 4 mL of phosphate buffer saline (PBS, pH 7.4) with 0.02% (wt/vol) sodium azide, was gently layered over the solidified gel in each vial. Vials were sealed with parafilm and maintained at 60 rpm in a 37°C water bath to replicate physiologic conditions. 2 mL out of the 4 mL of overlaid buffer solution was removed from each vial on days 1, 2, 3, 4, 5, 7, 10, and 14 and then weekly thereafter until no more IgG was released. Following collection, 2 mL PBS release buffer with 0.02% sodium azide (maintained at 37°C) was layered back into the test vials which were returned to the shaker bath until the next sampling period. The concentration of released IgG samples was evaluated by comparing the released samples to a standard IgG calibration curve. Calibrators and release samples (200 *μ*L) were pipetted into a 96-well, UV-free microplate (Greiner Bio-One, Monroe, NC) in triplicate and the absorbance was measured at 280 nm (Synergy 2 Microplate Reader, BioTek, Winooski, VT). All experiments were set up in triplicate and absorbance of a blank control (0% IgG in 22.5% PTS*gel*) was subtracted from the released samples for estimation of protein concentration. Similar experiments were set up using three different concentrations of 10GH PTS*gel* to evaluate further modulation of the release profiles.

### 2.6. After Releasing IgG Integrity

#### 2.6.1. Sodium Dodecyl Sulfate Polyacrylamide Gel Electrophoresis (SDS-PAGE) Analysis: Reducing and Nonreducing

Collected buffer samples from the* in vitro* release assays were evaluated for IgG integrity by SDS-PAGE analysis. The IgG samples included were standards and the samples released in PBS buffer (pH 7.4, 37°C) after incorporation into a selected PTS*gel*. Samples were evaluated within 7 days of collection and stored at 4°C until analysis. IgG standards, diluted in PBS, or sample eluates were combined with 4x Laemmli dye, with (reducing) or without (nonreducing) *β*-mercaptoethanol, to achieve a 1x dye concentration. Reduced samples were heated to 95°C for 10 minutes, cooled, and loaded on 4–12% Bis-Tris NuPAGE gels (Life Technologies, Carlsbad, CA). Prestained markers (cat. number LC5925, Life Technologies) and nonreduced samples were loaded without heating. Gels were run in MOPS buffer (Life Technologies), with antioxidants for reduced samples only, at 180 V for 60 minutes. Electrophoresed gels were fixed in 50% methanol, 10% acetic acid for 30 minutes, stained in Coomassie Brilliant Blue R250 Staining Solution (Bio-Rad Laboratories, Inc., Hercules, CA) for 30 minutes, and destained in 5% methanol, 7.5% acetic acid until clearance. Gels were scanned on a Canon CanoScan 9000F at 600 dpi and images saved as TIFFs in Adobe Photoshop.

#### 2.6.2. Size Exclusion HPLC Analysis (Shodex Column)

Collected buffer samples from the* in vitro* release assays were evaluated for IgG integrity by SE-HPLC analysis. The IgG samples included were standards and the samples released in PBS buffer (pH 7.4, 37°C) after incorporation into a selected PTS*gel* for up to 28 days. Samples were stored at −20°C until analysis. Twenty microliters of IgG standards, diluted in PBS, or sample eluates were analyzed on a Shodex column (KW403-4F, 4.6 mm ID × 300 mm L) using a UV absorption detector with the wavelength selected at 214 nm. The mobile phase was 100 mM sodium phosphate, 250 mM NaCl, pH 7.0, used under isocratic conditions at 0.35 mL/min.

### 2.7. *In Vivo* Subcutaneous Release of IgG in Mice

Use of animals in this study was approved and monitored by the North Carolina State University Institutional Animal Care and Use Committee (IACUC). IgG was labeled with a near-infrared (NIR) dye (IRDye 800CW by LICOR Biosciences, Lincoln, NE). NIR-labeled IgG in PTS*gel* gelling solution or in PBS was made by adding 1 mL of cold PTS*gel* (25% polymer solution in PBS, pH 7.4) or PBS to 1 mg of lyophilized NIR-labeled IgG. After gentle vortexing, the solutions were stored at 4°C until being used within 24 hours. Insulin syringes with 31 G needles were used to inject 200 *μ*L of solution subcutaneously over the dorsum of a female CD-1 mouse (10 weeks of age) (Charles River, Morrisville, NC) that was maintained on an alfalfa-free diet. Mice (*n* = 3) were injected with PBS, PTS*gel*, or a combination of NIR-IgG in PBS or NIR-IgG in PTS*gel*. Mice were anesthetized with 2.5% isoflurane in oxygen and imaged using an* in vivo* imager (IVIS, Xenogen, Alameda, CA) using Indocyanine Green (ICG) settings. Quantification of fluorescence was measured using the imaging software automatic region of interest (ROI) setting to calculate the radiant efficiency of the injection site. Mice were imaged prior to injection, immediately after injection, and then on days 1–5, 7, 10, and 14 after injection and then weekly using the same imager settings and protocol as used for day 0 imaging.

### 2.8. *In Vivo* Safety Assessment

Assessment of the injection site was done at each imaging time to evaluate for signs of inflammation or swelling. Once the injection site was negative for dye detection on IVIS imaging, the mice were euthanized and the skin at the injection site was collected, the inverted skin exposing the injection site/PTS*gel* depot was imaged* ex vivo* using IVIS imaging to detect residual IgG, and the skin section was fixed in 10% formalin. The formalin-fixed skin was then processed for histopathology, stained with hematoxylin and eosin, and examined using light microscopy.

## 3. Results 

### 3.1. PTS*gel* Synthesis

For the purposes of this study, five thermosensitive PTS*gel* polymers were synthesized. The five polymers developed for this study were designated 101GH, 10GH, 103GH, 113GH, and 122GH. The polymers were constructed with different block sizes of m-PEG, PCL, and PLA with PLA in the center of the molecule (m-PEG_*x*_-PCL_*y*_-PLA_*z*_-PCL_*y*_-PEG_*x*_-m). The molecular weight (Mw) ranged between 3000 and 4000 Da with gradual increase in the hydrophobicity of molecules. The objective was to vary molecular weights and hydrophobic-hydrophilic block ratios in the polymers to achieve modulation of drug release. In the described polymers, the PEG blocks were Mw of 500 or 550 Da, the PCL block Mw ranged from 400 to 800 Da, and the PLA block was of constant size at Mw 1100 across all polymers. All polymers were characterized by NMR, FTIR for structural confirmation, and GPC for PDI determination and ability to transition from liquid phase to gel at 37°C. All five polymers were compared for* in vitro* release profiles. Two polymers, 10GH and 113GH, were used for* in vivo* subcutaneous release, polymer disappearance, and safety investigations. 10GH at various concentrations was also analyzed for* in vitro* degradation analyses.

### 3.2. PTS*gel *Characterization

**Figure 1:**
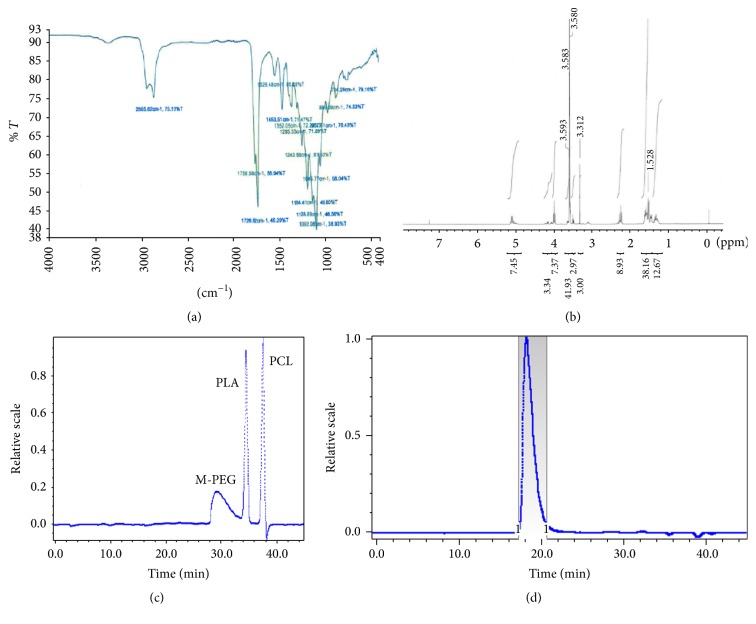
(a) FTIR spectrum of 10GH PTS*gel*. (b) ^1^H NMR spectrum of PEG_*x*_-PCL_*y*_-PLA_*z*_-PCL_*y*_-PEG_*x*_. (c) GPC chromatogram for reference standards (m-PEG, PCL, and PLA). (d) GPC chromatogram for 10GH PTS*gel*. PD of the 10GH polymer = 1.08.

*FTIR Spectrum*. FTIR spectrum of 10GH polymer is reported in Figure 1(a). Absorption band at 1726 cm^−1^ and multiple bands ranging from 1000 to 1300 cm^−1^ established the presence of ester linkages in pentablock copolymer. Existence of terminal hydroxyl group was confirmed by C-O stretching band at 1092 cm^−1^ and O-H band (stretch) in the range of 3300–3400 cm^−1^. C-H stretching bands at 2938 and 2866 cm^−1^ depicted presence of PCL blocks. The presence of N-H (stretch) in the urethane group may be overlapped in the area of O-H band (stretch) 3300–3400 cm^−1^. Similarly, the C=O in urethane group might have been overlapped with other C=O (stretch) peaks from ester linkages in pentablock copolymer. Absorption band at 1529 cm^−1^ (N-H bending) also exhibited the presence of urethane group in pentablock copolymer. 

^*1*^ *H-NMR Spectrum*. A Mercury 300 MHz NMR spectrometer was employed to characterize the pentablock copolymers.  Figure 1(b) depicts ^1^H-NMR spectra of 10GH in deuterated chloroform. As described in Figure 1(b), typical ^1^H-NMR characteristic peaks were observed at 1.55, 2.30, and 4.04 *δ* ppm representing methylene protons of -(CH_2_)_3_-, -OCOCH_2_-, and -CH_2_OOC- of PCL units, respectively. A sharp peak at 3.64 *δ* ppm was attributed to methylene protons (-CH_2_CH_2_O-) of PEG. Typical signals at 1.50 (-CH_3_) and 5.17 (-CH-) *δ* ppm were assigned for PLA blocks, whereas a peak at 3.36 *δ* ppm was denoted to terminal methyl (-OCH_3_-) of PEG. The [EO]-[CL]-[LA] molar ratios of final products were calculated from integrations of PEG signal at 3.36 *δ* ppm, PCL signal at 4.04 *δ* ppm, and PLA signal at 5.17 *δ* ppm. PEG signal at 3.36 *δ* ppm was applied for the calculation of molar ratio of various blocks within the pentablock copolymer. Estimated molecular weight, calculated using NMR, was close to theoretical feed ratio (Table 1). 

*Molecular Weight (Mw and Mn) and Polydispersity Determination*. Molecular weight (Mw and Mn) and polydispersity of polymers were determined by GPC. Retention time for the three components used for PTS*gel* synthesis is depicted in Figure 1(c). A typical GPC chromatogram of 10GH pentablock copolymer is shown in Figure 1(d). A single peak for the polymer was observed describing unimodal distribution of molecular weight and absence of any other homopolymer block such as PEG, PCL, or PLA. Polydispersity (PDI) for the five analyzed polymers ranged from 1.08 to 1.28 indicating narrow distribution of molecular weights. Estimated molecular weights of synthesized PTS*gel* were close to the feed ratio (Table 1). 

*Residual Tin, Organic Solvents, and Endotoxin Evaluation*. Each polymer was tested for tin levels, organic solvents, and endotoxin levels. Sample analysis for the determination of residual dichloromethane (DCM) and heptane was performed by GC-MS. In all samples, no residual heptane was detected. Residual DCM was 0.2 ppm (10GH), 124 ppm (101GH), 74 ppm (103GH), 170 ppm (113GH), and 278 ppm (122GH). Stannous (tin) levels were less than 2 ppm. Endotoxin levels were determined to be less than 0.1 EU/mL.

### 3.3. PTS*gel* Gelling Evaluation

**Figure 2:**
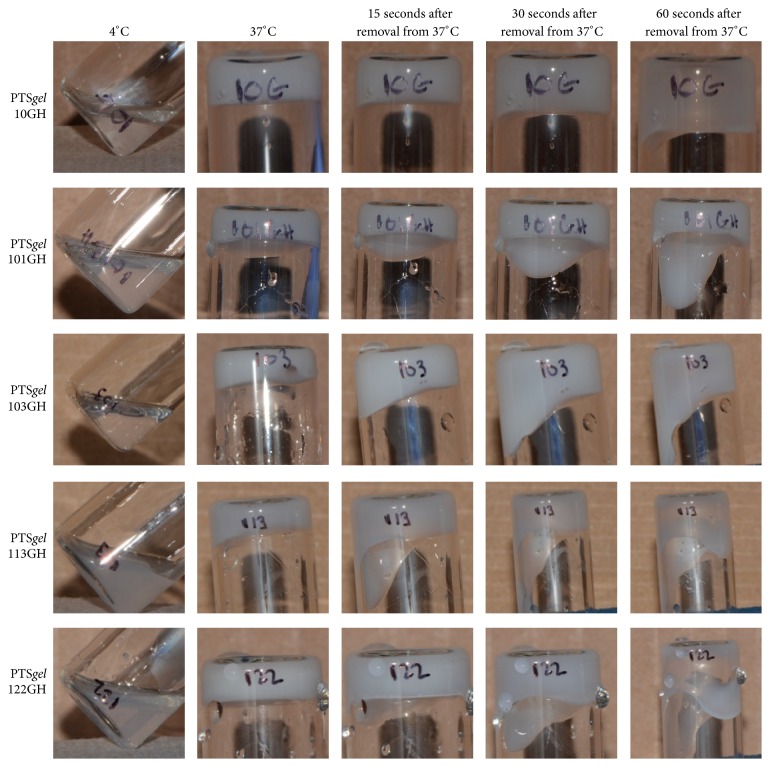
Sol-gel transition of 10GH, 101GH, 103GH, 113GH, and 122GH. At 4°C temperature the PTS*gels* are in liquid form and transition into solid gels at 37°C. The gels slowly transition back to liquid form at room temperature.

All polymers were free flowing liquids at 4°C. Sol-gel transition for the five polymers is as shown in Figure 2. The PTS*gels* 10GH and 103GH were clear liquids, whereas the PTS*gels* 113GH and 122GH were slightly turbid liquid at 4°C (Figure 2). Immediately after removal of the vials from the 37°C water bath, the PTS*gels* were a solid, slightly opaque white gel. On inversion of gel tubes at room temperature, the PTS*gels* remained a solid gel for 15 to 60 seconds (Figure 2). Eventually, all gels slowly transitioned back to liquid form at room temperature. All five polymers could be injected through a 31-gauge needle and form a solid gel in PBS at 37°C (see videos in Supplementary Material available online at http://dx.doi.org/10.1155/2016/2407459).

### 3.4. *In Vitro* PTS*gel* Disintegration (Gravimetric Measurement)

**Figure 3:**
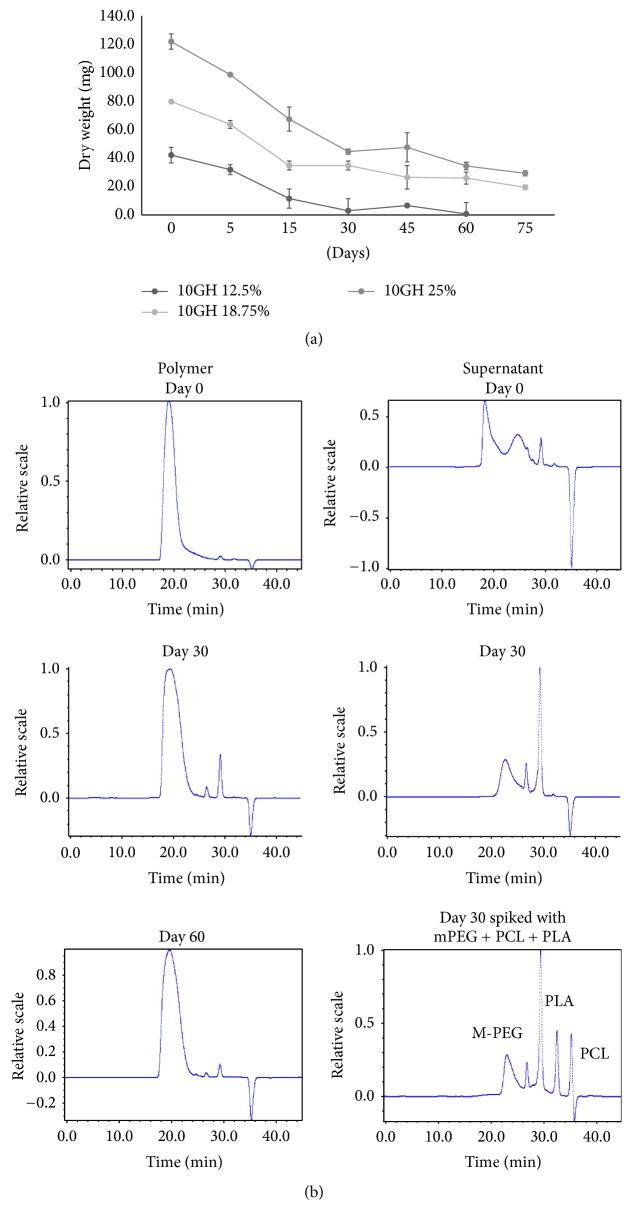
(a)* In vitro* dissolution/disintegration of 10GH PTS*gel*. Three concentrations (12.5, 18.75, and 25%) of PTS*gel* 10GH (500 *μ*L in triplicate) were evaluated for disintegration in phosphate buffered saline (pH 7.4)* in vitro* at 37°C. On day 0 and after 5, 15, 30, and 45 days and every 15 days thereafter, overlaid buffer was removed and the remaining PTS*gels* were lyophilized and weighed to determine PTS*gel* dry weight over time. (b) GPC analysis of residual polymer (10GH, 25%) and supernatant collected on various days for* in vitro* disintegration in PBS buffer, pH 7.4 at 37°C.

The gravimetric dry weight loss over time of PTS*gel* 10GH (12.5, 18.75, and 25%) in PBS was evaluated at 37°C. There was a large difference in dry weight between the* PTSgel* concentrations at time 0, with 25% polymeric gel having a mean weight of 120.8 mg, 18.75% polymer of 79.1 mg, and 12.5% polymer of 41.8 mg. The lowest concentration of 10GH (12.5% gelling polymer) nearly completely disintegrated by 30 days (92.7%), while 18.75 and 25% 10GH PTS*gel* demonstrated disappearance of polymer with 74.2 and 76% dissolved, respectively, by day 75 (Figure 3(a)).

Residual polymer and supernatant collected at various time points were analyzed by GPC (Figure 3(b)). It is visually clear that the polymer peaks eluted as a symmetrical peak at day 0 and at days 30 and 60. However, the peak at days 30 and 60 is wider than that observed at day 0 suggesting degradation of the polymer. The supernatant shows a small amount of dissolved polymers on day 0; however, there is no polymer present on day 30 and the disintegrated fragments are much smaller than the polymer but larger than the monomers used for the synthesis. The individual peaks have not yet been further characterized.

### 3.5. Modulation of* In Vitro* IgG Release from PTS*gels*

**Figure 4:**
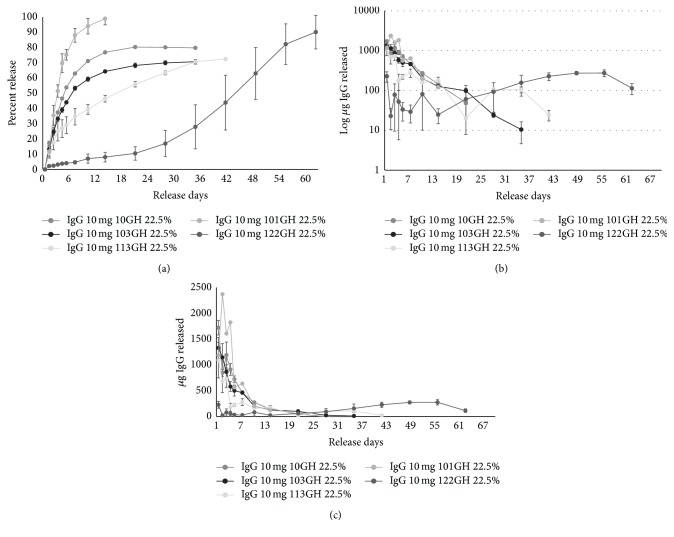
*In vitro* release of IgG from pentablock copolymers (PTS*gel*). Mean ± SD of cumulative release (a) and daily release of IgG from PTS*gel* on a log scale (b) and on a linear scale (c). Using 20 mg/mL IgG in 22.5% PTS*gel* maintained at 37°C, half of overlaid PBS buffer was removed and replaced at days 1, 2, 3, 4, 5, 7, 10, and 14 and then weekly thereafter.

**Figure 5 fig5:**
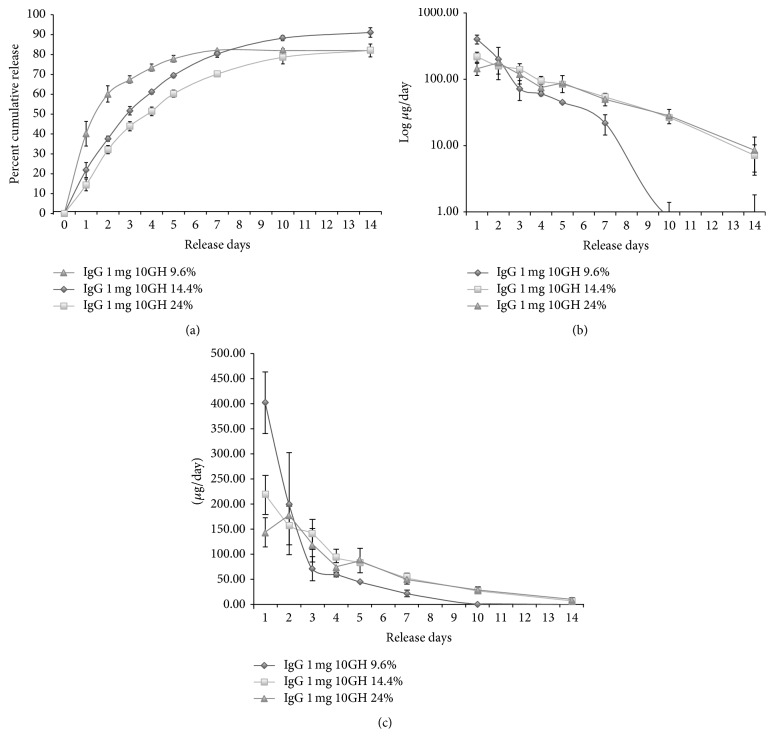
Effect of polymeric gel concentration on* in vitro* release of IgG from pentablock copolymers (PTS*gel*). Mean ± SD of cumulative release (a) and log (b) and linear (c) daily release of IgG from PTS*gel*.

The solid gel PTS*gel* was maintained at 37°C and half of PBS buffer was removed and replaced on days 1, 2, 3, 4, 5, 7, 10, and 14 and then weekly thereafter until no additional release was observed. Extensive modulation of* in vitro* release was achieved by using five different PTS*gels*, 101GH, 10GH, 103GH, 113GH, and 122GH, with increasing hydrophobicity of the polymers in the order, respectively. In most polymers, there was a low initial burst of drug release at 20 mg/mL IgG (11–24% release on day 1) and an almost negligible release with 122GH (~2%), the most hydrophobic polymer. Initial release is followed by a controlled release over extended period of several days to weeks as shown in Figure 4. Using 22.5% of each PTS*gel*, the quickest release rate was observed with the most hydrophilic polymer in the group (101GH) with 100% of the IgG load released in 14 days. The PTS*gels* 10GH and 103GH had a slower release with 80% and 69% of the IgG released in 21 days, respectively. Both the PTS*gels* 113GH and 122GH had a much slower rate of release with 73% and only 44% by day 42, respectively. From 122GH, 91% of IgG release was achieved by day 63 (Figure 4(a)). A significant decrease in initial burst release related to increase in hydrophobicity demonstrates that the capacity to hold drugs possibly increases with increase in hydrophobicity of these PTS*gels*. Also, modulation of drug release at different rates can be achieved for long periods by changing the hydrophobicity of the polymers, as shown in Figures 4(b) and 4(c). As the polymer became more hydrophobic, the drug was released at a slower rate and the drug release had a much longer duration. The initial burst is also reduced considerably when lower IgG concentrations (2 mg/mL) were loaded into the polymer, as is shown in Figure 5 for PTS*gel* 10GH, suggesting that the polymer's capacity to hold the drug may dictate initial burst release.

In addition,* in vitro* release of IgG was also demonstrated to be modulated by varying the concentration of PTS*gel* polymer. Using 2 mg/mL IgG in 10GH PTS*gel*, 9.6% PTS*gel* resulted in a faster release of IgG, accompanied by a higher (40%) initial release on day 1 and 80% cumulative release by day 7, compared to a gradual and more sustained release achieved with 14.4 and 24% of 10GH PTS*gel* and less initial burst (Figure 5), again strongly suggesting that the drug release is a function of polymer capacity which increases with higher polymer concentration. Optimal loading for each therapeutic at different gelling polymer concentration will need to be determined during development.

### 3.6. Structural Integrity of IgG Released from PTS*gel*

**Figure 6:**
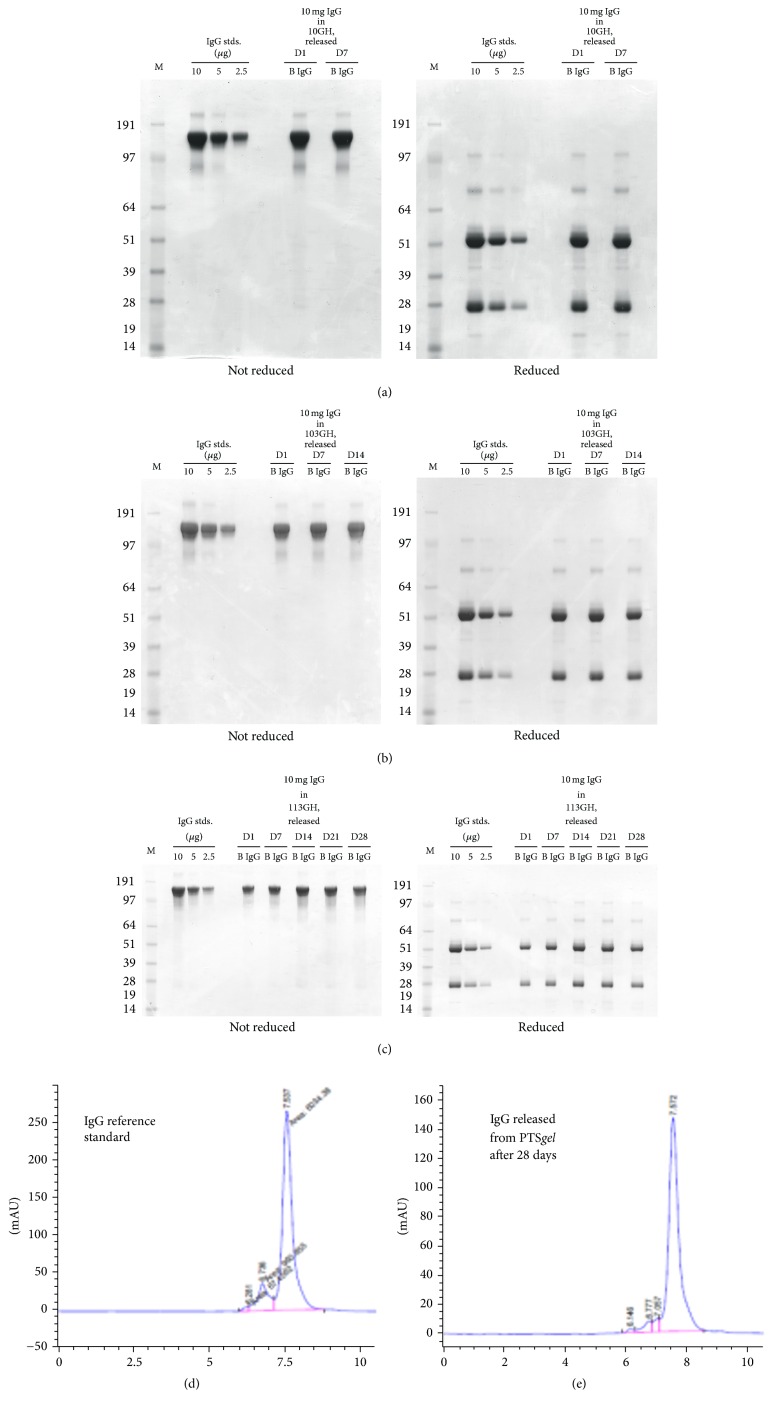
IgG integrity evaluated by SDS-PAGE. Reduced (using beta-mercaptoethanol) and nonreduced sodium dodecyl sulfate-polyacrylamide gel electrophoresis (SDS-PAGE) were used for size-based separations of the IgG for determining IgG integrity in the PTS*gel in vitro* release rate samples over time. The IgG released from 10GH (a), 103GH (b), and 113GH (c) had the same bands of approximately 150 kDa for the nonreduced and 28 and 51 kDa for the reduced SDS-PAGE at each time point tested, which appeared identical to the IgG standards. D, release day; B, blank lanes; M, molecular size marker; IgG stds., IgG standards. (d) and (e) SE-HPLC analysis of IgG reference standard (d) and of released sample (e) after incubation with PTS*gel* 10GH for 28 days in PBS at 37°C.

*(A) Using SDS-PAGE Analysis.* Reduced (using beta-mercaptoethanol) and nonreduced sodium dodecyl sulfate-polyacrylamide gel electrophoresis (SDS-PAGE) were used for size-based separations of the IgG for determining IgG integrity in the* in vitro* samples released from PTS*gel* over a period of seven days. Reducing conditions disrupt disulphide bonds separating IgG light and heavy chains. The IgG released from 10GH had the same bands of approximately 150 kDa for the nonreduced and 28 and 51 kDa for the reduced SDS-PAGE at days 1 and 7, which appeared identical to the IgG standards (Figure 6(a)). These results suggest that the IgG molecules were intact and no degradation of IgG had occurred during incubation with PTS*gel* and after release into the buffer. Similarly, there appeared to be excellent integrity for the IgG released from the 103GH polymer through 14 days (Figure 6(b)) and the 113GH polymer through 28 days (Figure 6(c)), suggesting that the PTS*gel* polymer did not affect the integrity of the IgG protein. Low amounts of PLA utilized in the synthesis of PTS*gel* polymers do not result in lowering local pH upon polymer degradation and hence offer safe environment to drugs, especially biologics, for sustained release. 

*(B) SE-HPLC Analysis*. Figures 6(d) and 6(e) compare chromatograms of SE-HPLC analysis conducted on a reference standard IgG and on an* in vitro* sample released after incubation with 10GH for 28 days at 37°C. IgG maintained its structural integrity as is clearly demonstrated in Figures 6(d) and 6(e).

### 3.7. *In Vivo* Studies for IgG Release

**Figure 7:**
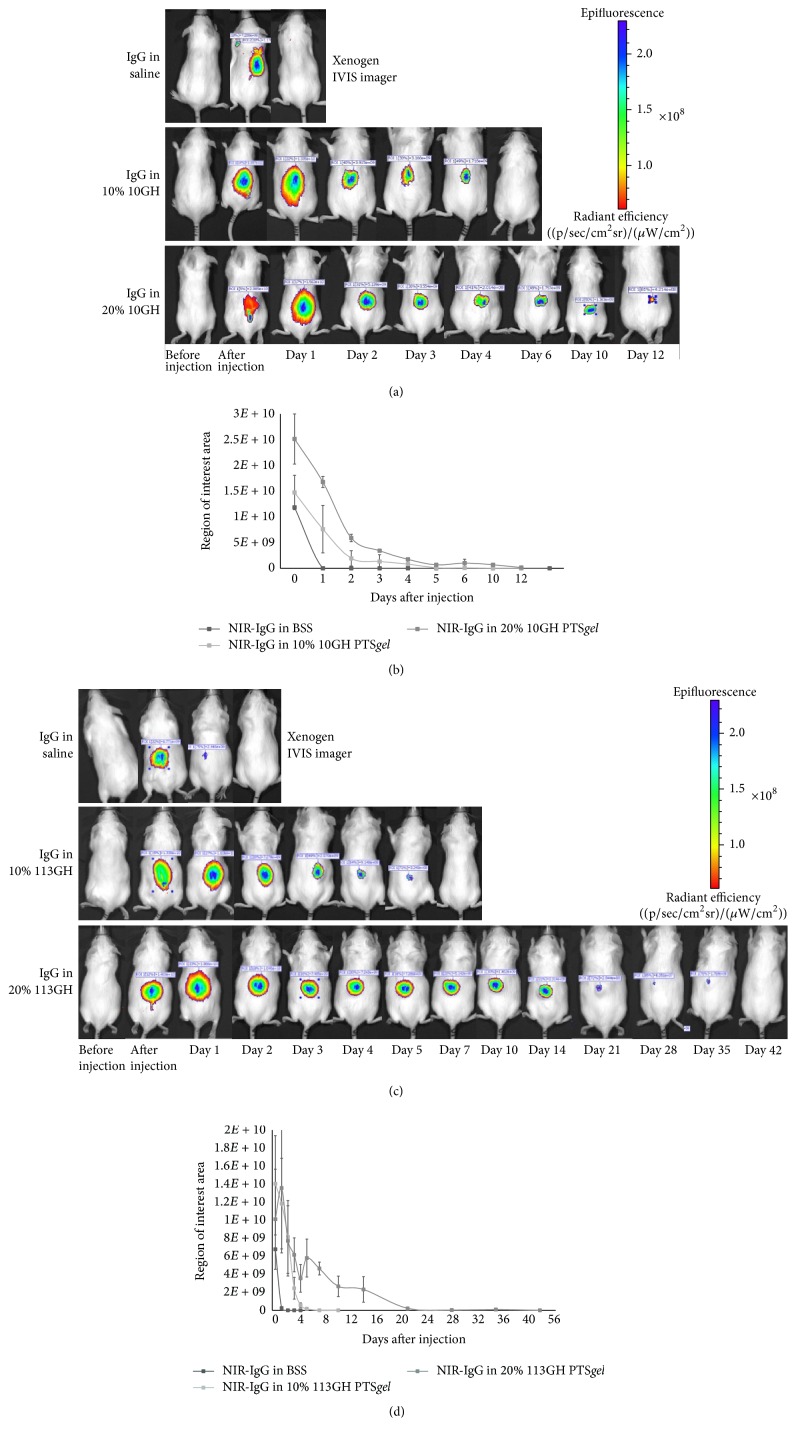
*In vivo* imaging of mice after subcutaneous injection of PTS*gel*. Mice were injected with PBS, PTS*gel* (10% and 20% 10GH or 113GH), or a combination of NIR-IgG PBS or NIR-IgG PTS*gel* in triplicate. Mice were anesthetized and imaged prior to injection, immediately after injection, and then on days 1–5, 7, 10, and 14 after injection and then weekly thereafter using an* in vivo* imager (IVIS, Xenogen, Alameda, CA) ((a) and (c)). Quantification of fluorescence was measured using the imaging software automatic region of interest (ROI) setting to calculate the radiant efficiency of the injection site ((b) and (d)).

Following subcutaneous injection of 200 *μ*L of PTS*gel* (either 10GH or 113GH) containing 200 *μ*g of near-infrared dye labeled IgG (NIR-IgG) or NIR-IgG in PBS in mice, no adverse reaction, swelling, or redness was observed at the injection site for the duration of the study. NIR-IgG in PBS was visible on IVIS imaging immediately after injection but was no longer visible 24 hours after injection (Figures 7(a)–7(d)). Fluorescence of NIR-IgG in the PTS*gel in vivo* paralleled the* in vitro* release rates for 10GH and 113GH presented in Figure 4. Mice injected with 10% 10GH had fluorescence through approximately day 4, while those injected with 20% 10GH fluoresced through approximately day 14 (Figures 7(a) and 7(b)). Mice injected with 10% 113GH fluoresced through approximately 7 days, and those injected with 20% 113GH fluoresced through approximately 35 days (Figures 7(c) and 7(d)).

**Figure 8:**
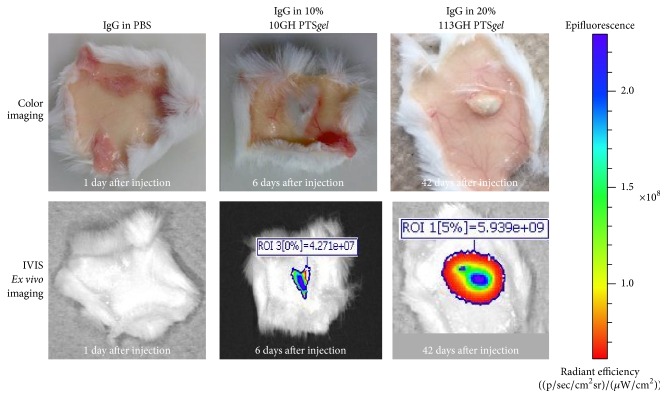
*Ex vivo* imaging of mice skin after subcutaneous injection of PTS*gel*. Once the mice were negative for fluorescence on IVIS imaging, they were euthanized; the skin at the site of the injection was excised and imaged* ex vivo*.

Once negative for fluorescence on IVIS imaging, mice were euthanized; the skin at the site of the injection was excised and imaged* ex vivo*. In all animals, there was a very small deposit of gel visible in the subcutaneous tissue, indicating the nearly complete dissolution/disintegration of the PTS*gel* (Figure 8). On imaging, there was a small signal of fluorescence that corresponded to approximately the size of the gel deposit, suggesting that IgG was tightly held by the PTS*gel* and that IgG release and gel dissolution/disintegration occurred in parallel and that an “empty shell” of undissolved PTS*gel* did not remain.

### 3.8. *In Vivo* Safety Assessment after Subcutaneous Injection of NIR-IgG with PTS*gel*

**Figure 9:**
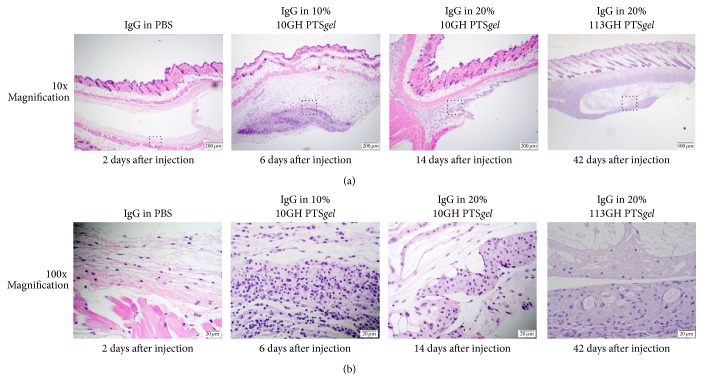
Dermal histopathology after subcutaneous injection of PTS*gel*. Using lower magnification light microscopy (a), the subcutaneous depot of PTS*gel* could be visualized in the 10 and 20% 10GH- and 113GH-injected tissue. Dotted box in (a) was the site of higher magnification represented in (b).

Following imaging, skin samples were fixed and processed for histopathology. When examined using light microscopy, the subcutaneous depot of PTS*gel* could be visualized and was mildly infiltrated with mononuclear cells. At the 6-day examination of the 10% PTS*gel* 10GH, there were a few scattered neutrophils and macrophages surrounding and infiltrating the site of the injection. At 14 days with the 20% PTS*gel*, there were only a few macrophages associated with the gel deposit. At 42 days with the 113GH PTS*gel*, there were macrophages surrounding the depot but no epidermal or dermal inflammation or swelling was observed (Figure 9).

## 4. Discussion

We have demonstrated, both* in vitro* and* in vivo*, that PTS*gels* can modulate the release of IgG, a model therapeutic protein, from less than 7 days to greater than 63 days and possibly much longer by altering block composition for change in hydrophobicity and/or aqueous polymer solution concentration as well as amount of drug loaded in the polymer. These polymers seem to exhibit a very high capacity to hold IgG and thus result in minimal initial burst. The exact capacity for each polymer is currently undergoing investigation in our laboratory. We also demonstrated that the IgG was associated with the PTS*gel* as* in vitro* release and* in vivo* degradation of polymers occurred in parallel. In addition, we demonstrated that the PTS*gel* provides a safe environment for the protein as evidenced by the intact IgG structure when tested on day 28 of* in vitro* release from the PTS*gel*. Since IgG structure remained intact after release from PTS*gel* and based on work in progress in our laboratory, we anticipate that the functional proteins would also maintain their bioactivity when incubated with PTS*gel*. The PTS*gel* composition and concentration determined timing of gel dissipation* in vivo*. Only small remnants of PTS*gel* were present* ex vivo* in subcutaneous tissues of mice at the time of euthanasia and these gel remnants had NIR-labeled IgG visible on IVIS imaging, suggesting that the rate of IgG release and gel disappearance occurred in parallel. In a separate study, a similar phenomenon was observed for NIR-labeled IgG when injected intracamerally [15]. The 25% PTS*gel* (10GH) gradually dissolved/disintegrated over the course of 30 days in the anterior chamber of the eye and the PTS*gel* had a strong signal of NIR-IgG in small remnants of PTS*gel* until the gel was completely dissolved or disintegrated [15].

When evaluating the* in vitro* IgG release, an initial 1-day to 2-day higher release (burst effect) was observed with lower concentrations of PTS*gel* (9.6% polymer), while in the higher concentrations of PTS*gel* (22 or 24% polymer), reduced burst effects were observed (see Figures 4 [Fig fig5]–6) and they exhibited sustained release of IgG which paralleled the polymer dissolution/disintegration (see Figure 3), suggesting that the IgG was not just physically entrapped in the gel but perhaps a large portion of IgG is likely entrapped in the micellar structure of the polymer [12] which is released as the polymer dissolves or disintegrates. We have also observed this phenomenon with proteins other than IgG (data not shown). This suggests that initial burst effect likely occurs when added drug exceeds the capacity of the PTS*gel* to hold the drug for sustained release. As stated earlier, the dose capacity, though it has not been measured quantitatively, is likely dependent on a combination of factors such as polymer composition and percentage of the PTS*gel*.

In this study we achieved extensive modulation of IgG release from 7 days to longer than 63 days by varying the Mw size of PCL block comprising the PTS*gel.* Release rates of IgG from the fastest to the slowest (i.e., 101GH (fastest release) to 122GH (slowest release)) correlated with higher hydrophobicity created by increasing the PCL block size (from Mw 400 to 800). As shown in Figure 4, the PTS*gel* 122GH shows a continuing IgG release at 63 days, suggesting an expected duration of* in vitro* IgG release of 70 to 80 days. The PTS*gel* 113GH achieved sustained IgG release for at least 42 days (mean 278 *μ*g (day 7) to 25 *μ*g released on day 42), PTS*gel* 103GH for at least 21 days, and PTS*gel* 10GH for at least 14 days (each from a 22.5% polymer and total loading of 10 mg IgG) (see Figure 4). Patel et al. [13] demonstrated similar release of IgG with PTS*gel* 113GH but required the use of polymer-IgG nanoparticles to achieve a 45-day release profile, which is far shorter duration than what we have been able to obtain with PTS*gel* 122GH alone.

Although we demonstrated* in vivo* that IgG remained in the gel until the polymer was nearly completely dissolved, further studies are needed to confirm drug bioactivity after delivery and to evaluate pharmacokinetics and pharmacodynamics for each route of therapy. However, a steady release* in vitro* which correlated well to the decrease in NIR-IgG* in vivo* was observed; hence one can hypothesize that* in vivo* drug release may be similar to that observed* in vitro*. Further studies are ongoing in our laboratory to evaluate drug bioactivity and to demonstrate pharmacokinetics and pharmacodynamics from protein and small molecule drug release of both hydrophobic and hydrophilic nature from a variety of PTS*gels*.

## 5. Conclusions

PTS*gels* create biocompatible/biodegradable thermosensitive formulations which can provide sustained release of biologics without chemical modification of the therapeutic molecule. Modulation of release and control of initial burst of IgG, a model therapeutic protein, was achieved by varying block composition and aqueous solution concentration of the PTS*gels*. When injected subcutaneously, PTS*gels* provided a safe environment for the protein and were well tolerated. Importantly, disintegration of the polymer paralleled IgG release* in vivo*, leaving little or no remnant of PTS*gel* after drug release was complete.

## Supplementary Material

Aqueous solutions (25%) of pentablock co-polymers at 4°C were injected through a 31-gauge needle into a container of 37°C PBS. Video demonstrating the immediate gelling properties of 10GH (A), 103GH (B), 113GH (C), 122GH (D), and 101GH (E) are shown.

## Figures and Tables

**Table 1:** Mw, Mn, and PDI determination of PTS*gel*s.

PTS*gel* ID	Total Mn^a^ (theoretical)	Total Mn^b^ (calculated)	Total Mn^c^ (calculated)	Mw^c^ (GPC)	PDI^c^
101GH	3000	3898	4784	5132	1.07
10GH	3100	3534	4855	5264	1.08
103GH	3600	4271	3404	4347	1.28
113GH	3500	3542	4615	5078	1.1
122GH	3700	3941	4349	4941	1.14

^a^Theoretical value, calculated according to the feed ratio.

^b^Calculated from ^1^H NMR.

^c^Determined by GPC analysis.
