# Supplementary material for: Sustained Release of Protein Therapeutics from Subcutaneous Thermosensitive Biocompatible and Biodegradable Pentablock Copolymers (PTSgels)
Source: J Drug Deliv. 2016 Oct 5;2016:2407459. doi: 10.1155/2016/2407459 (PMC5069382; doi:10.1155/2016/2407459)
Supplement: Supplementary file 1 — Aqueous solutions (25%) of pentablock co-polymers at 4°C were injected through a 31-gauge needle into a container of 37°C PBS. Video demonstrating the immediate gelling properties of 10GH (A), 103GH (B), 113GH (C), 122GH (D), and 101GH (E) are shown. [file 2407459.f1.pptx]

## Slide 1
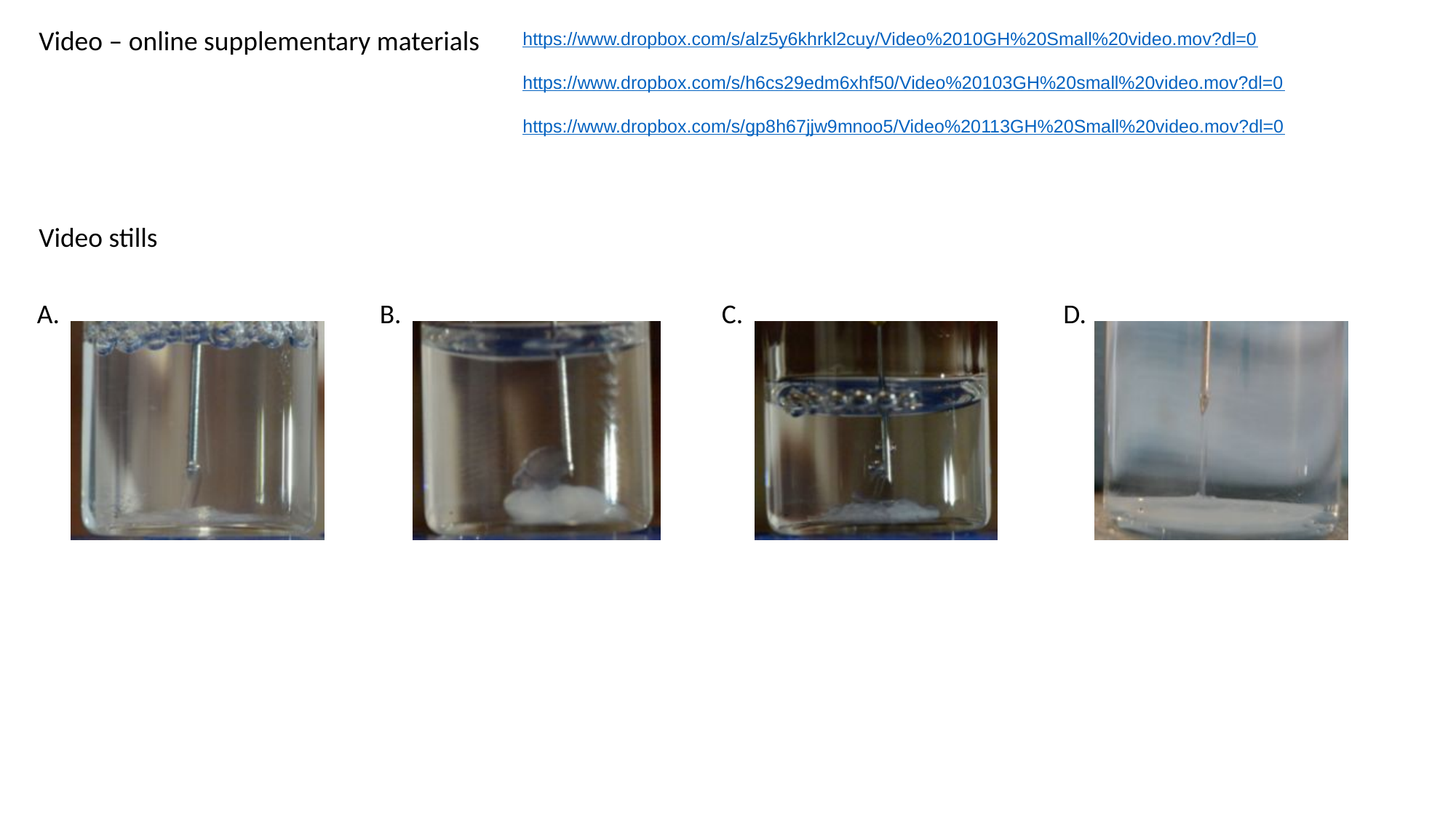

https://www.dropbox.com/s/alz5y6khrkl2cuy/Video%2010GH%20Small%20video.mov?dl=0
https://www.dropbox.com/s/h6cs29edm6xhf50/Video%20103GH%20small%20video.mov?dl=0
https://www.dropbox.com/s/gp8h67jjw9mnoo5/Video%20113GH%20Small%20video.mov?dl=0
Video – online supplementary materials
Video stills
A.
B.
C.
D.

## Slide 2
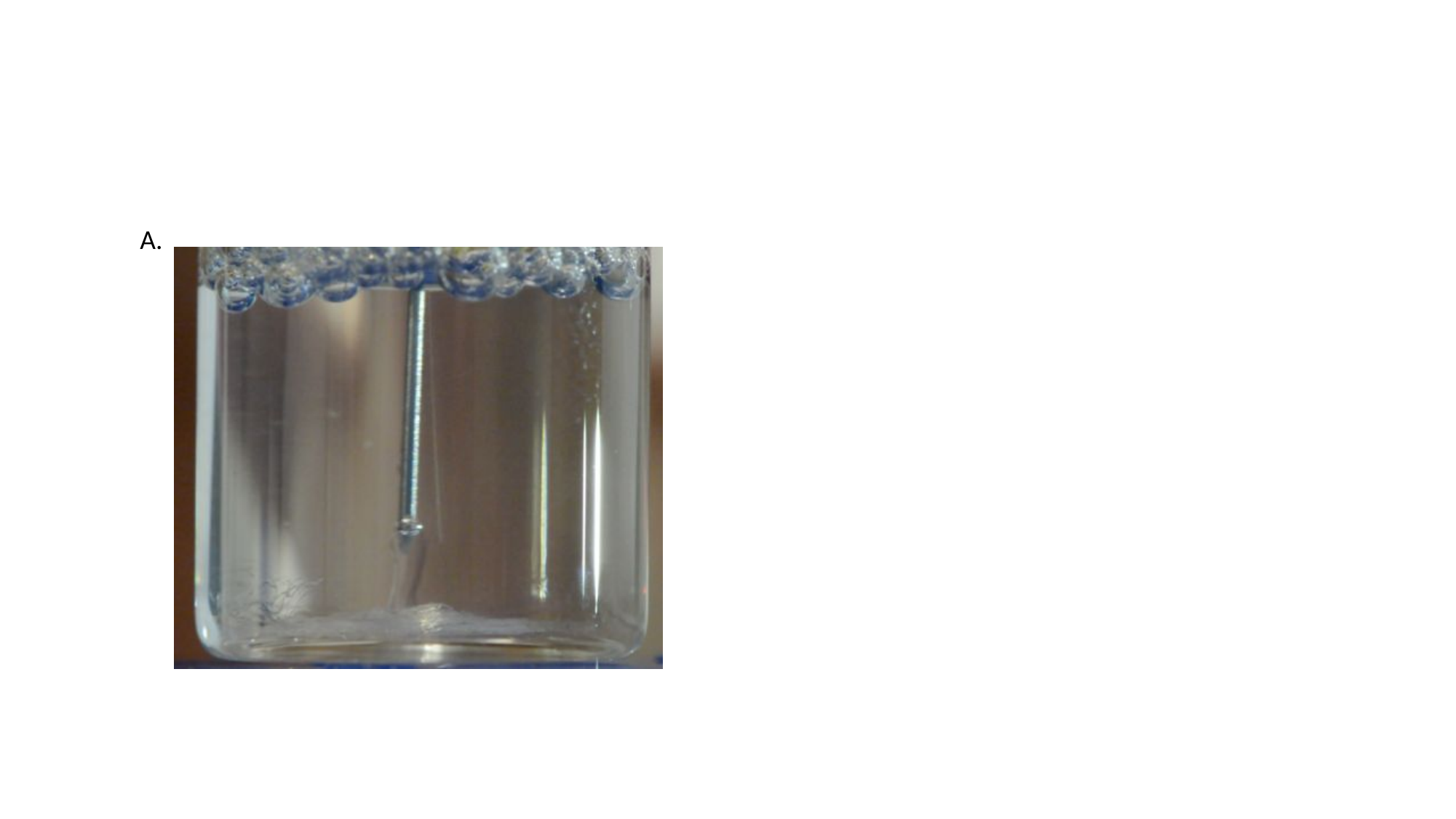

A.

## Slide 3
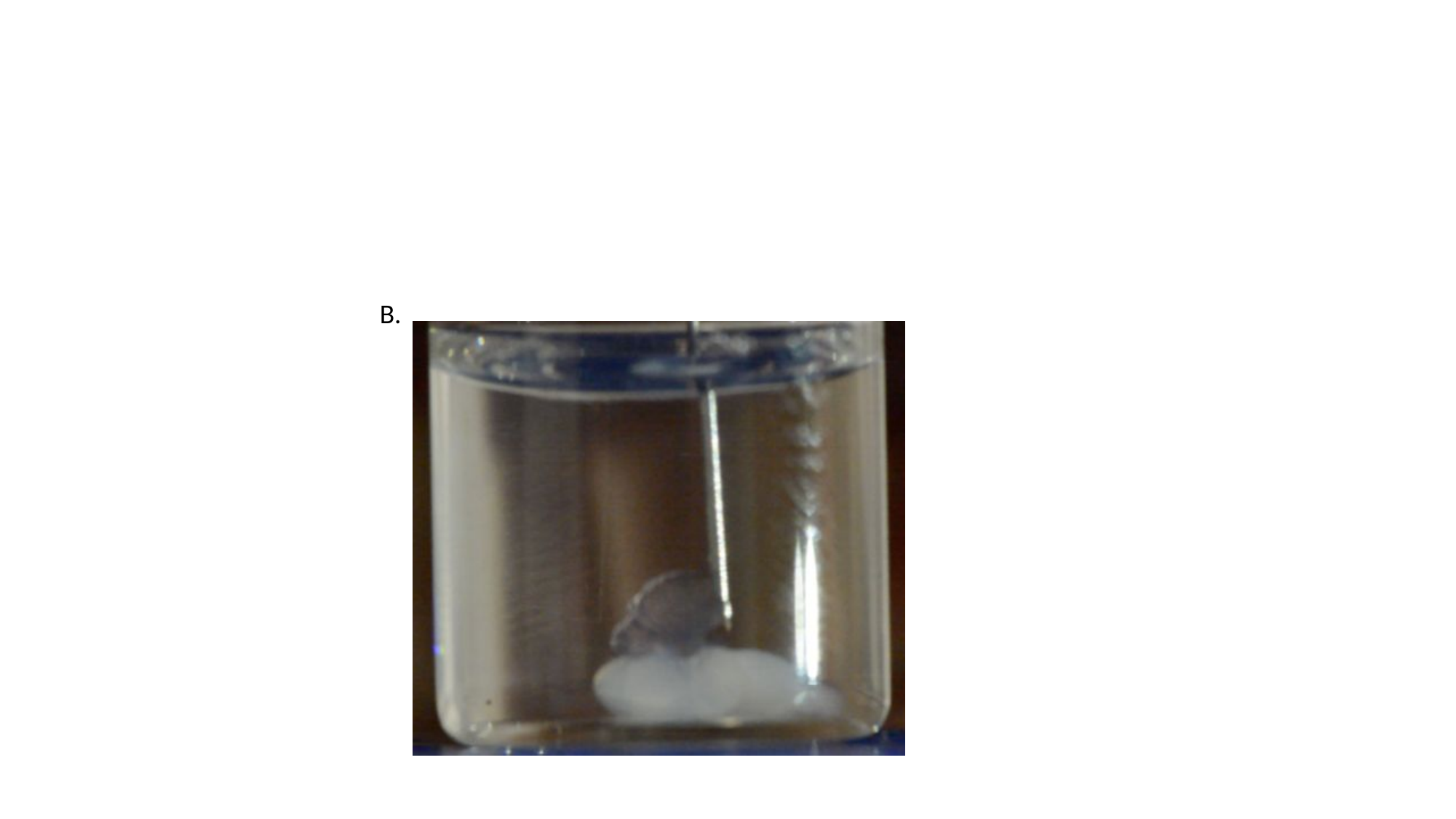

B.

## Slide 4
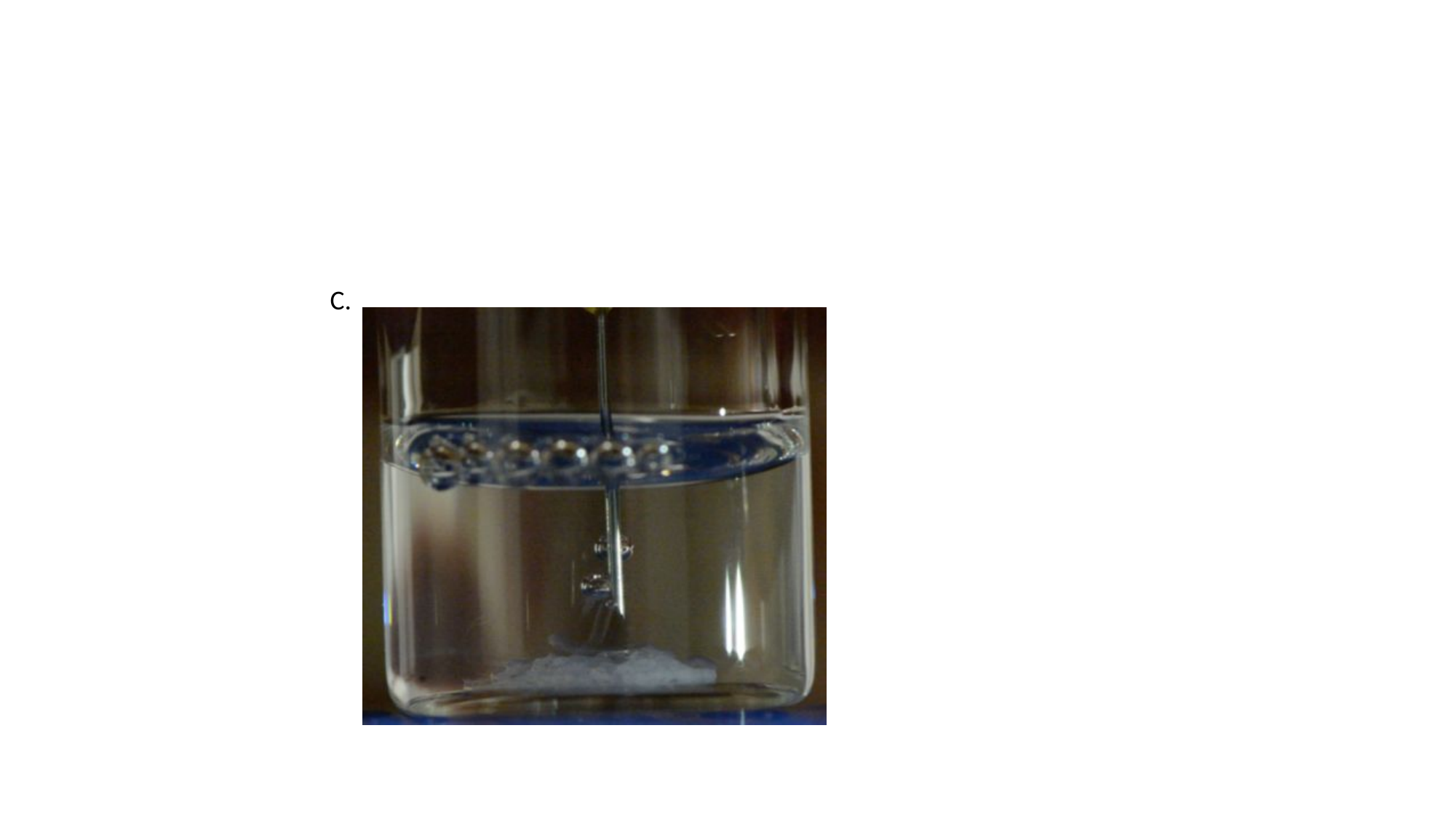

C.

## Slide 5
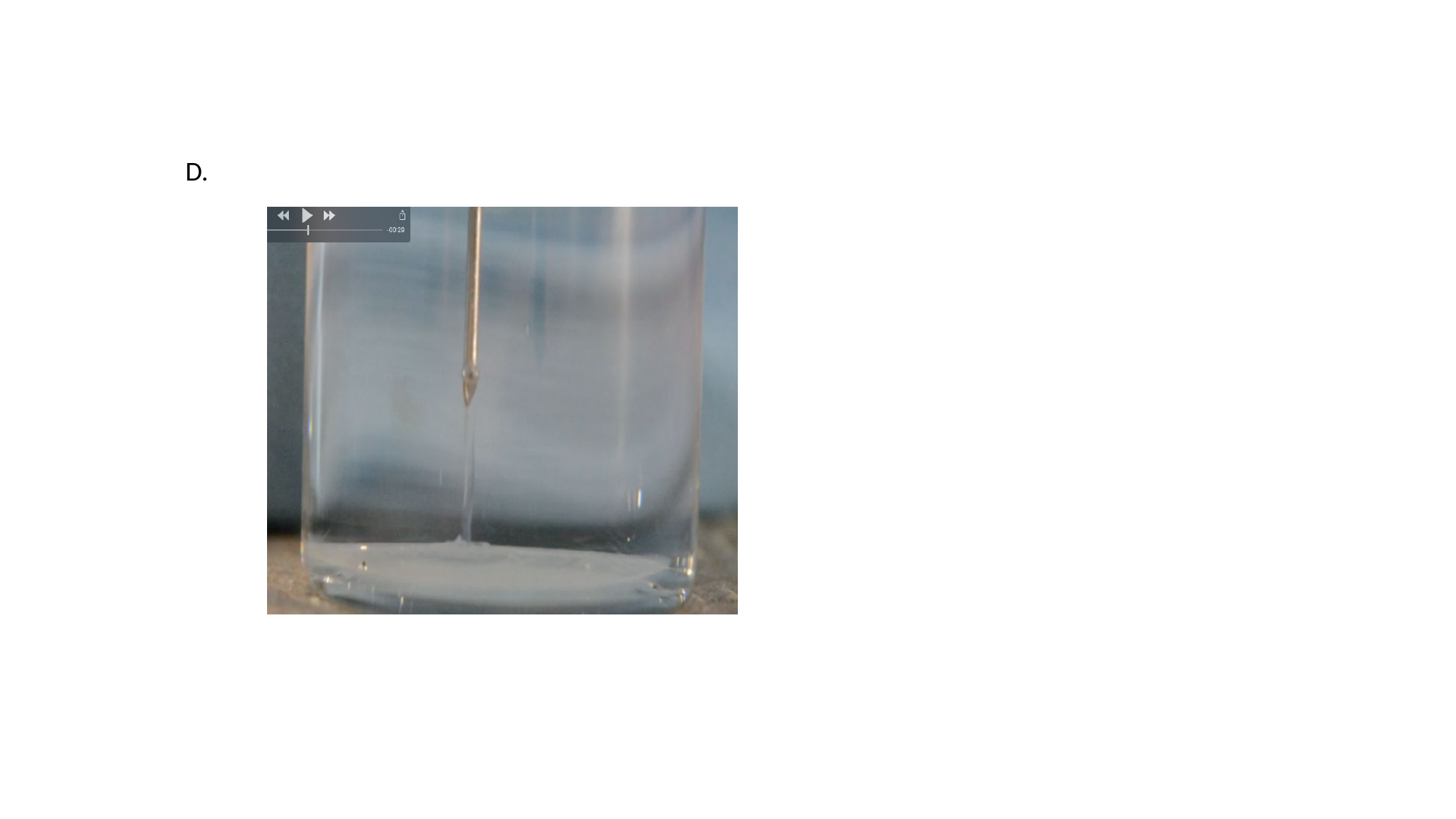

D.
